# Spatial Regulation of Mitochondrial Heterogeneity by Stromal Confinement in Micropatterned Tumor Models

**DOI:** 10.1038/s41598-019-47593-8

**Published:** 2019-08-01

**Authors:** Hydari Masuma Begum, Hoang P. Ta, Hao Zhou, Yuta Ando, Diane Kang, Kristen Nemes, Chelsea F. Mariano, Jia Hao, Min Yu, Keyue Shen

**Affiliations:** 1 Department of Biomedical Engineering, Viterbi School of Engineering, University of Southern California, Los Angeles, CA 90089 USA; 2 Department of Stem Cell Biology and Regenerative Medicine, Keck School of Medicine, University of Southern California, Los Angeles, CA 90033 USA; 3 Mork Family Department of Chemical Engineering, Viterbi School of Engineering, University of Southern California, Los Angeles, CA 90089 USA; 4 Norris Comprehensive Cancer Center, Keck School of Medicine, University of Southern California, Los Angeles, CA 90033 USA; 5 USC Stem Cell, Keck School of Medicine, University of Southern California, Los Angeles, CA 90033 USA

**Keywords:** Breast cancer, Cancer microenvironment, Tumour heterogeneity, Cancer models, Cancer metabolism

## Abstract

Heterogeneity of mitochondrial activities in cancer cells exists across different disease stages and even in the same patient, with increased mitochondrial activities associated with invasive cancer phenotypes and circulating tumor cells. Here, we use a micropatterned tumor-stromal assay (μTSA) comprised of MCF-7 breast cancer cells and bone marrow stromal cells (BMSCs) as a model to investigate the role of stromal constraints in altering the mitochondrial activities of cancer cells within the tumor microenvironment (TME). Using microdissection and RNA sequencing, we revealed a differentially regulated pattern of gene expression related to mitochondrial activities and metastatic potential at the tumor-stromal interface. Gene expression was confirmed by immunostaining of mitochondrial mass, and live microscopic imaging of mitochondrial membrane potential (ΔΨ_m_) and optical redox ratio. We demonstrated that physical constraints by the stromal cells play a major role in ΔΨ_m_ heterogeneity, which was positively associated with nuclear translocation of the YAP/TAZ transcriptional co-activators. Importantly, inhibiting actin polymerization and Rho-associated protein kinase disrupted the differential ΔΨ_m_ pattern. In addition, we showed a positive correlation between ΔΨ_m_ level and metastatic burden *in vivo* in mice injected with MDA-MB-231 breast cancer cells. This study supports a new regulatory role for the TME in mitochondrial heterogeneity and metastatic potential.

## Introduction

Despite the latest advances in cancer therapeutics, the five-year survival rate for patients diagnosed with metastatic breast cancer is at a staggering 27% as opposed to 99% for those with localized disease^1^. A better understanding of metastatic progression is therefore crucial to improve prevention and treatment of advanced breast cancer.

Mitochondria have recently emerged as a potential regulator of cancer progression and metastasis. It has been shown that cancer cells depend on mitochondrial respiration for their *in vivo* tumor-forming ability^2–4^, and that mitochondrial metabolites play a role in driving oncogenesis^5^ and epithelial-mesenchymal transition (EMT)^6^, a phenotypic switch that precedes metastasis^7^. Importantly, there is significant heterogeneity in mitochondrial phenotypes across cancer disease stages, and even in the same patient. Increased mitochondrial redox activities in tumors have been correlated with tumor aggressiveness and metastatic potential^8^. Higher mitochondrial membrane potential (ΔΨ_m_) is associated with cancer cell survival and invasiveness^9–12^. In breast cancer, circulating tumor cells (CTCs), the presumptive precursor of metastases, exhibit enhanced mitochondrial biogenesis and respiration compared to cancer cells from primary tumors in the same host^13^. However, questions regarding where and how the heterogeneity of mitochondrial activities arises, and its impact on metastatic development, remain unanswered.

The tumor microenvironment (TME) plays an important role in cancer progression and metastasis^14^. The TME of progressing breast tumors is often characterized by distinct architectural and cytological features, including an evolving tissue interface of direct tumor-stromal interactions^15–18^ and a stiffening tumor mass^19^. Recently, it was reported that some stromal cells can regulate metabolic and/or mitochondrial functions in cancer cells through paracrine growth factor signaling and metabolite exchange^20,21^, or through transfer of mitochondrial DNA into cancer cells^3,4^. On the other hand, biomechanical properties of the TME have also been found to influence cancer cell invasiveness and metastatic potential^19,22^. At the tissue level, mechanical stresses in solid tumors are spatially dependent on tumor architecture and growth^23^. At the cellular level, mechanical cues are involved in regulating cancer cell proliferation^24^, invasiveness^25^, and extracellular matrix (ECM) remodeling^26,27^. Although not yet reported in cancer cells, it has been shown that mechanical stimuli can affect mitochondrial activity in cardiomyocytes and endothelial cells^28^, and that cytoskeletal remodeling leads to changes in mitochondrial dynamics^29^. However, it remains unclear whether stromal cells and their associated mechanical cues within the tumor architecture can drive heterogeneous mitochondrial activities.

We have previously established a micro-engineered tumor model, i.e., a micropatterned tumor-stromal assay (μTSA), to demonstrate that tumor-stromal interactions within the architectural context of a tumor play an important role in inducing phenotypic heterogeneity in cancer and stromal cells *in vitro*, which was confirmed *in vivo*^17^. In this report, we use the µTSA to investigate the role of stromal cells in regulating tumor mitochondrial heterogeneity *in vitro*. We reveal that ΔΨ_m_, mitochondrial mass, metabolism, and metastatic potential are spatially distributed within the µTSA. We show that ΔΨ_m_ levels correlate with YAP/TAZ nuclear translocation status, are regulated by stromal confinement, and are dependent on the actin cytoskeleton. We further demonstrate a positive correlation between ΔΨ_m_ levels and metastatic potential in cancer cells *in vivo*. We demonstrated in the µTSA a new regulatory role that the TME plays in mitochondrial heterogeneity and metastatic potential.

## Results

### Mitochondrial and metastatic pathways are upregulated at the tumor-stromal interface in the µTSA

**Figure 1:**
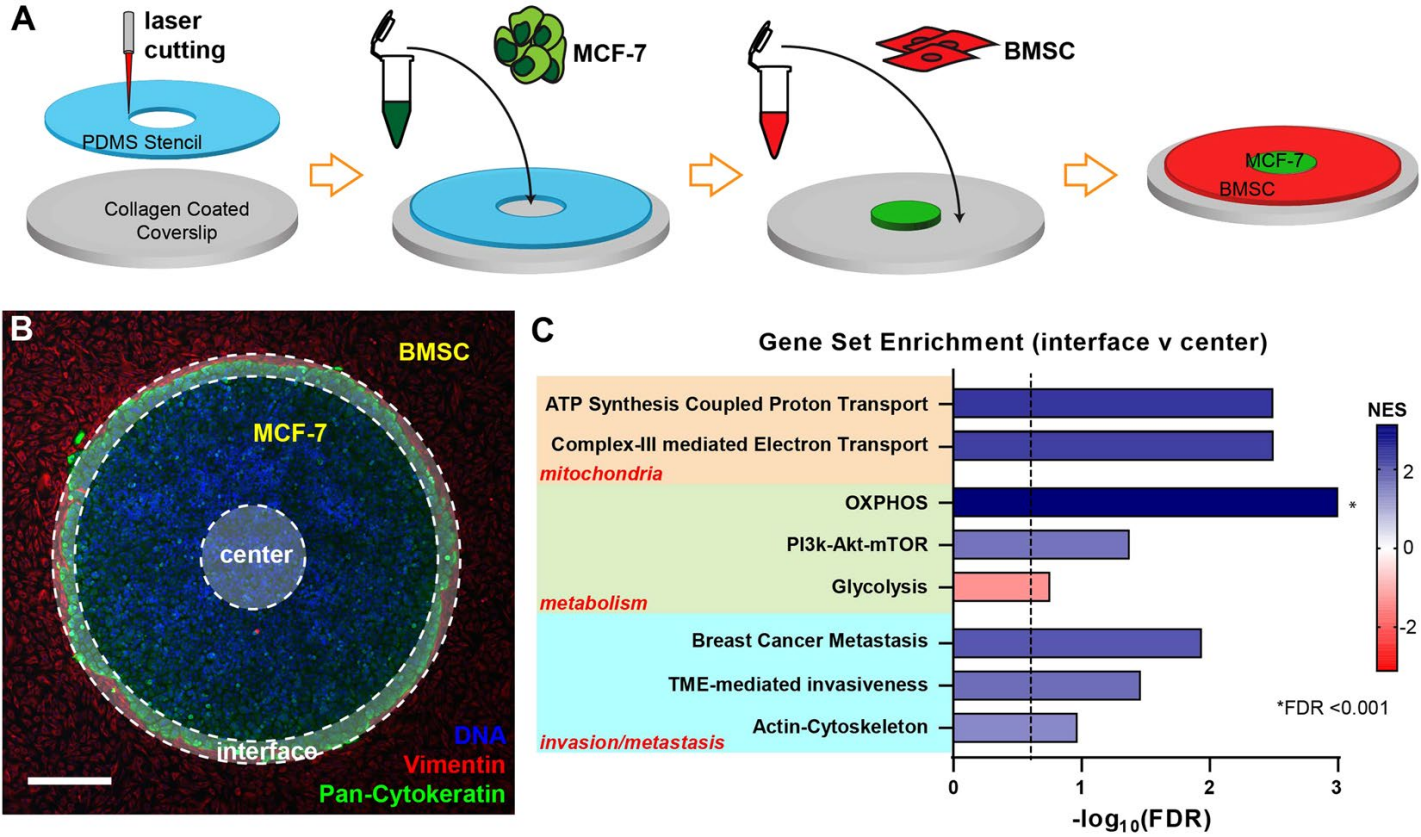
Spatial regulation of mitochondrial, metabolic, and metastatic pathways in micropatterned tumor-stromal assays (µTSA) assessed by RNA sequencing. (**A**) Schematics depicting the steps to create a MCF-7/BMSC µTSA; (**B**) Representative micropattern of an MCF-7 island (green: Pan-Cytokeratin) surrounded by BMSCs (red: Vimentin). Blue: nuclei. Scale bar: 100 μm. Cancer cells from the center and edge of the µTSA (opaque annular ring with dotted outlines) were isolated by laser capture microdissection^17^, and their RNA was extracted and sequenced; (**C**) Gene sets found to be enriched at the interface vs. center by RNA sequencing and gene set enrichment analysis (glycolysis has negative NES indicating enrichment in the center). (See Methods for more details. FDR: False Discovery Rate; FDR < 0.25 was considered significant.) MCF-7 cells from >6 micropatterns/experiment were microdissected and combined before RNA isolation and sequencing; N = 2 independent experiments.

To recapitulate cell-cell interactions within the tumor architectural constraints of the TME^17^, MCF-7 breast cancer cells were cultured in the µTSA with surrounding bone marrow stromal cells (BMSC)^30^ (Fig. 1A), resulting in adjacent spatial compartments of cancer and stromal cells with a defined tumor-stromal interface (Fig. 1B). To examine whether cancer cell phenotypes and signaling were differentially regulated at the transcriptome level, we used laser-capture microdissection (LCM) to obtain cancer cells from the interfacial and central regions of the µTSA island, and performed RNA sequencing (RNA-seq) on these cells. The RNA-seq data were analyzed by gene set enrichment analysis (GSEA), where statistical values were generated through the Wald Test, and gene sets were ranked by statistical significance and fold change direction (see Methods). Among the pathways that differ between the interface and center, we identified three categories gene sets among the 8 that were of interest in this study (Fig. 1C), i.e., 1) mitochondrial proton/electron transport activities; 2) cellular metabolism; and 3) cancer invasion and metastasis^31–38^. Specifically, genes related to mitochondrial activities were highly dependent on the cell location in the µTSA, with cells at the interface exhibiting upregulation of the electron and proton transport genes. At the metabolic level, interfacial cancer cells had upregulation of genes involved in oxidative phosphorylation (OXPHOS), contrary to cells at the center of the micropattern that showed upregulation of glycolysis genes. Consistent with the OXPHOS upregulation, genes from the PI3K-Akt-mTOR pathway^39^ were found to be upregulated at the interface. Notably, gene sets involved in actin-cytoskeleton and TME-mediated invasiveness were also upregulated at the interface, suggesting a role of the interface in cancer cell mechanics and migration. Importantly, a gene set that has been found to predict metastasis in lymph node-negative ER + breast cancer^37^ was also upregulated at the interface (Fig. 1C), matching the ER + subtype of MCF-7. The results here demonstrate spatially regulated heterogeneity of gene sets for mitochondrial activities, metabolism, and metastatic potential in cancer cells in the µTSA.

### Mitochondrial membrane potential (ΔΨ_m_) is spatially regulated in the µTSA

**Figure 2:**
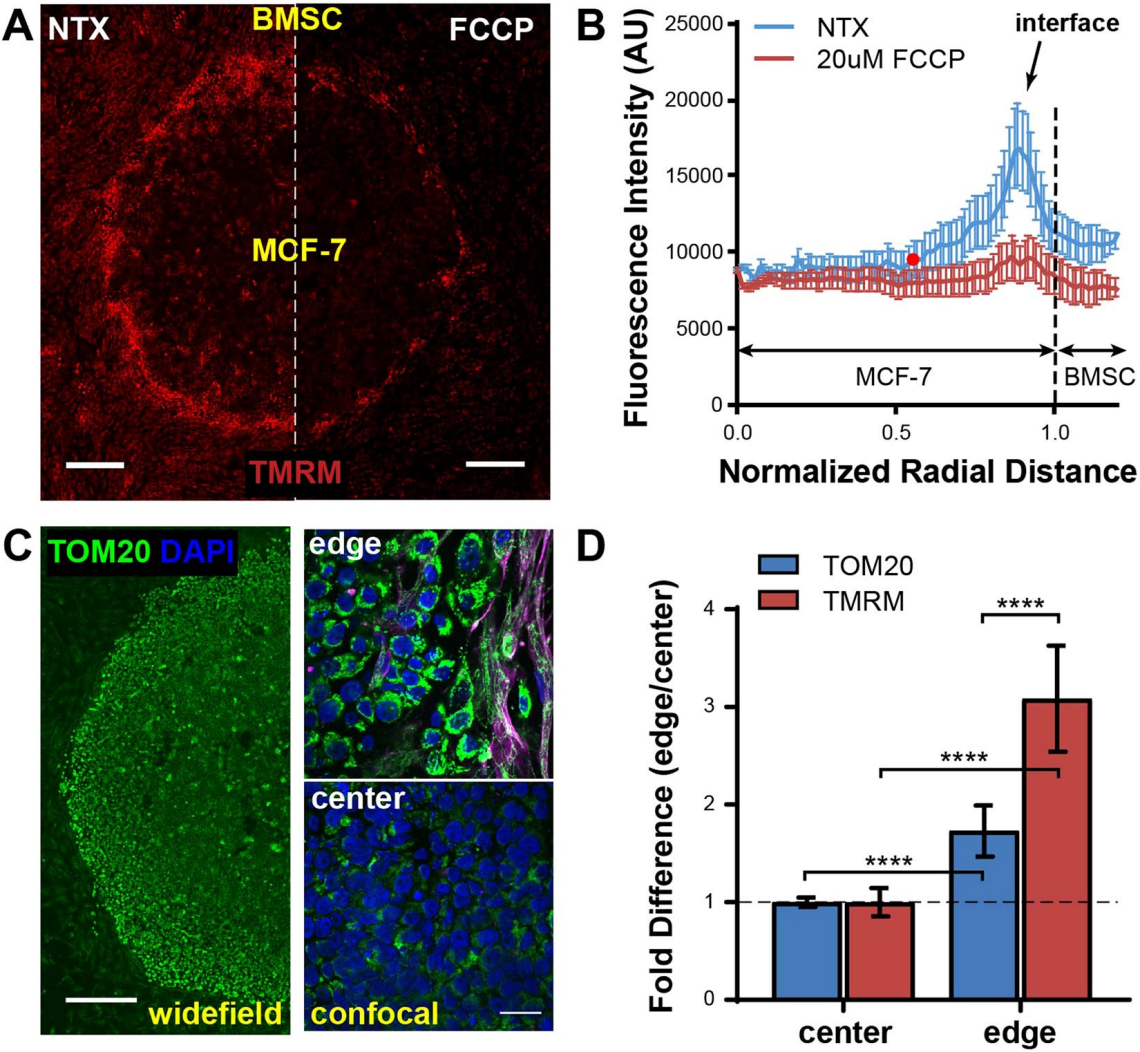
Differential regulation of mitochondrial membrane potential (ΔΨ_m_) and mass at the interface vs. center in the µTSA. (**A**) ΔΨ_m_ levels assessed by TMRM fluorescence on Day 4 MCF-7-BMSC µTSA before and after addition of 20 μM FCCP uncoupler. Scale bar: 500 μm; (**B**) Representative radial distribution of TMRM fluorescence in a MCF-7-BMSC µTSA on day 4 before and after FCCP treatment. The red dot (r = 0.52) on the no-treatment (NTX) curve indicates that the TMRM fluorescence to the right of the dot is significantly higher than that of all FCCP-treated samples; from N = 6 independent experiments (p < 0.05, Welch’s t-test); (**C**) µTSA stained for mitochondrial mass with anti-TOM20. Right panels: confocal scans of the edge and the center. Green: TOM20; purple: vimentin; blue: DAPI. Scale bars: 500μm in widefield (left) and 25μm in confocal (right). (**D**) Fold difference in TMRM and TOM20 fluorescence between the edge and the center. N = 3 independent experiments. P-values: ordinary one-way ANOVA.

To confirm that the mitochondrial proton/electron transport pathway is upregulated in the µTSA, we stained cells with tetramethylrhodamine methyl ester (TMRM), a reversible mitochondrial membrane potential (ΔΨ_m_) dye, which only accumulates in active mitochondria with intact membrane potential^40^. As shown in Fig. 2A, the ΔΨ_m_ of MCF-7 cells in the µTSA had a distinct spatial profile, with lower ΔΨ_m_ in cells at the center of the tumor micropattern, and higher in those closer to the interface. To validate that the TMRM signal is reflective of ΔΨ_m_, we treated the micropatterns with the ΔΨ_m_ uncoupler, carbonyl cyanide-4-(trifluoromethoxy)phenylhydrazone (FCCP)^41^. The FCCP treatment led to TMRM fluorescence loss in all MCF-7 cells (Fig. 2A,B). To quantitatively analyze the spatial regulation, we plotted the radial distribution of TMRM fluorescence within these micropatterns, where the normalized radial distances of 0 and 1 indicate the approximate center and edge of the micropatterned tumor island, respectively, and distances greater than 1 represent the surrounding stromal cell areas (Fig. 2B). TMRM fluorescence in MCF-7 cells within the micropattern was significantly higher than the residual TMRM fluorescence post-FCCP treatment at radial distances greater than 0.52 (as indicated by the red point on the no-treatment (NTX) curve in Fig. 2B).

Next, we investigated whether the differences in ΔΨ_m_ were due to a difference in mitochondrial mass in the cells at the center and interface. On Day 4 of µTSA cell culture, we fixed and immunostained cells for TOM20, a protein expressed on the mitochondrial outer membrane and commonly utilized for mitochondrial quantification^42^ (Fig. 2C). The fluorescence intensities of TOM20 and TMRM staining at the interface were both normalized against those at the center of the µTSA. We found that both mitochondrial mass and ΔΨ_m_ were enhanced at the interface compared to the center, but that the enhancement in ΔΨ_m_ (~3.1 fold) was significantly higher than that of the mitochondrial mass (~1.7 fold) (Fig. 2D). The results indicate that the ΔΨm increase at the interface is mainly due to increased proton gradient, and not to increased mitochondrial mass.

### Fluorescence microscopy of metabolic coenzymes reveals enhanced redox activity at the interface

**Figure 3:**
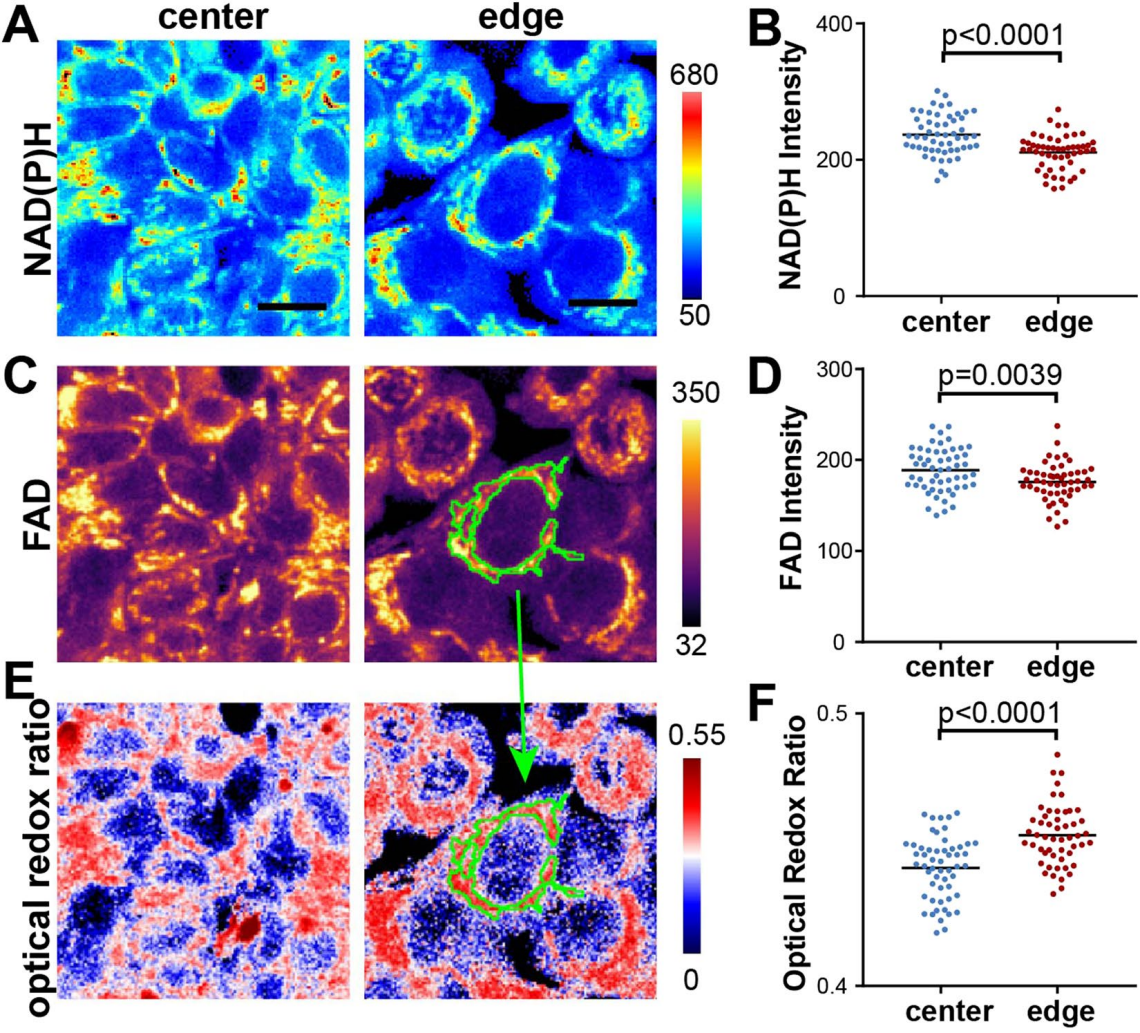
Redox imaging of cancer cells in a µTSA. Representative images of (**A**) NAD(P)H fluorescence; (**C**) FAD fluorescence; and (**E**) optical redox ratio (defined as FAD/(FAD + NAD(P)H)) at the center and edge of the µTSA on day 4. Scale bar: 25 μm. Quantification of (**B**) NAD(P)H and (**D**) FAD fluorescence intensities, and (**F**) the optical redox ratio at the single-cell level from the center (blue dots) or the edge (red dots) areas within the µTSA. Mitochondrial regions are segmented from FAD images and applied to the optical redox ratio images (green regions in **C**,**E**). Color scale is to the right of each image. (Representative dataset from N = 4 independent experiments; p-values: Welch’s t-test.)

To examine the differential metabolic functions in the µTSA, we employed a non-invasive, label-free approach, using microscopic imaging of the naturally occurring auto-fluorescence of the metabolic coenzymes, nicotinamide adenine dinucleotide (NADH) and its phosphate ester NADPH, designated NAD(P)H as their fluorescence cannot be distinguished^43,44^ (Fig. 3A,B), as well as flavin adenine dinucleotide (FAD) (Fig. 3C,D). The fluorescence of FAD and NAD(P)H was simultaneously acquired through 540/50 nm and 460/80 nm emission filters with a single two-photon excitation laser light source at 780 nm. The fluorescence signals were then processed to obtain the optical redox ratio (ORR), which is indicative of OXPHOS metabolism^45^, and is defined as the ratio of FAD fluorescence over the sum of signal intensity from FAD and NAD(P)H (Fig. 3E). The images were further segmented based on FAD intensity for mitochondrial regions^46^ at the subcellular level to specifically measure the ORR in mitochondria in individual cells (Fig. 3F). We observed altered energy metabolism in the mitochondria of interfacial cancer cells indicated by their higher ORR when compared to cells at the center of the µTSA (Fig. 3F). The results are consistent with the RNA-seq data showing spatially elevated mitochondrial OXPHOS at the µTSA tumor-stromal interface.

### ΔΨ_m_ spatial profile correlates with YAP/TAZ nuclear localization in the micropatterns

**Figure 4:**
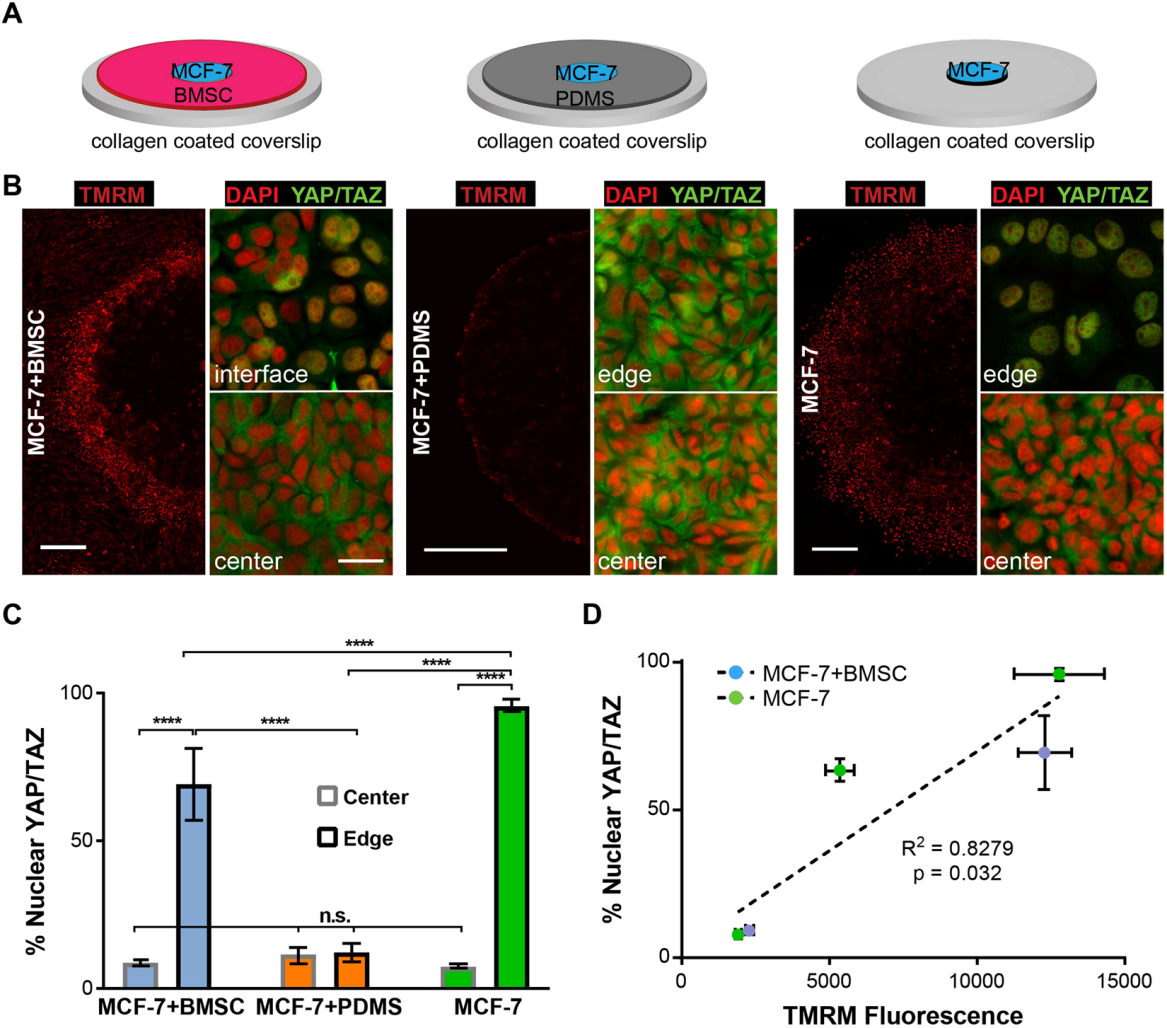
Correlation of ΔΨ_m_ and YAP/TAZ nuclear translocation in micropatterns. (**A**) Schematics of the three micropattern cultures used in this experiment; (**B**) TMRM staining of ΔΨ_m_ (scale bar: 500 μm) and YAP/TAZ immunostaining (scale bar: 25 μm) in the three micropatterns on day 4; (**C**) Quantification of cancer cells with nuclear YAP/TAZ localization at the center and edge of the µTSA (n.s.: not significant; ****p < 0.0001 by ordinary one-way ANOVA); Representative dataset from N = 3 independent experiments. (**D**) Linear regression of YAP/TAZ nuclear localization and TMRM fluorescence in cancer cells in the monoculture and co-culture µTSA. Three locations (center, edge and approximately 700 μm away from the edge) were taken from the monoculture µTSA and two locations (center and edge) from the co-culture. (Representative dataset, N = 2 independent experiments; p-value: zero-slope hypothesis in linear regression).

We then aimed to investigate the basis for the spatial differences in ΔΨ_m_ within the micropatterns. We had observed a significant upregulation of actin-cytoskeleton related genes at the tumor-stromal interface (Fig. 1C), which led us to hypothesize that the spatial patterns of ΔΨ_m_ in the µTSA result from the differential cellular constraints experienced by the epithelial cancer cells within the tumor island. Cells at the interface are weakly confined by stromal cells, whereas those closer to the center undergo heavier constraints from the neighboring epithelial cells. To test this hypothesis, in addition to the regular MCF-7-BMSC µTSA, we micropatterned MCF-7 islands surrounded by a thin layer of micro-contact printed polydimethylsiloxane (PDMS) to impose complete physical constraints on the island, as well as MCF-7 islands without stromal cells or PDMS, which lack epithelial or stromal constraints (Fig. 4A). Using TMRM staining, we found distinct differences in the spatial distribution of ΔΨ_m_ within the MCF-7 islands in the three micropatterns (Fig. 4B). MCF-7-BMSC µTSA showed higher ΔΨ_m_ in MCF-7 cells closer to the interface, consistent with results in Fig. 2. Constraining the tumor island with PDMS led to an almost complete loss of high ΔΨ_m_ at the micropattern edge. However, when the micropatterned tumor island grew free of any physical constraints, a wider band of cells with high ΔΨ_m_ was observed at the periphery of the micropattern (Fig. 4B).

To get a better molecular understanding of the ΔΨ_m_ regulation, we immunostained the micropatterns for the transcriptional regulators, YAP/TAZ, which can translocate from the cytosol to the nucleus, reflecting contact-mediated Hippo-signaling^47,48^ or mechanical cues experienced by the cells^49,50^. Figure 4B shows representative images of subcellular localization of YAP/TAZ at the center and edge of the micropatterns. The percentage of cells with nuclear YAP/TAZ in these regions was calculated (Fig. 4C). We found that MCF-7 cells at the edges of the co-culture or of the monoculture had higher levels of nuclear YAP/TAZ when compared to their respective centers. When surrounded by PDMS, YAP/TAZ localization across the whole micropattern became predominantly cytoplasmic, as seen at the center of the mono- and co-cultures (Fig. 4B,C). Interestingly, the percentage of nuclear YAP/TAZ in MCF-7 cells at the edge of co-cultures was intermediate and significantly different from those at the edges of the monoculture and PDMS-confined controls, suggesting that stromal cells impose an intermediate level of physical constraints. Next, we plotted nuclear YAP/TAZ vs. TMRM fluorescence from the monoculture and co-culture conditions. We picked three locations (edge, center, and intermediate) from the monoculture and two locations (edge and center) from the co-culture based on the visible differences in TMRM intensities among these regions. Interestingly, we found a strong positive correlation between the two parameters (p = 0.032, R^2^ = 0.8279, Fig. 4D). Together, the results suggest that the spatial distribution of ΔΨ_m_ of cancer cells in micropatterns stems from the differential physical constraints imposed on cells.

### ΔΨ_m_ distribution and YAP/TAZ nuclear translocation in µTSA are modulated by stromal density

**Figure 5:**
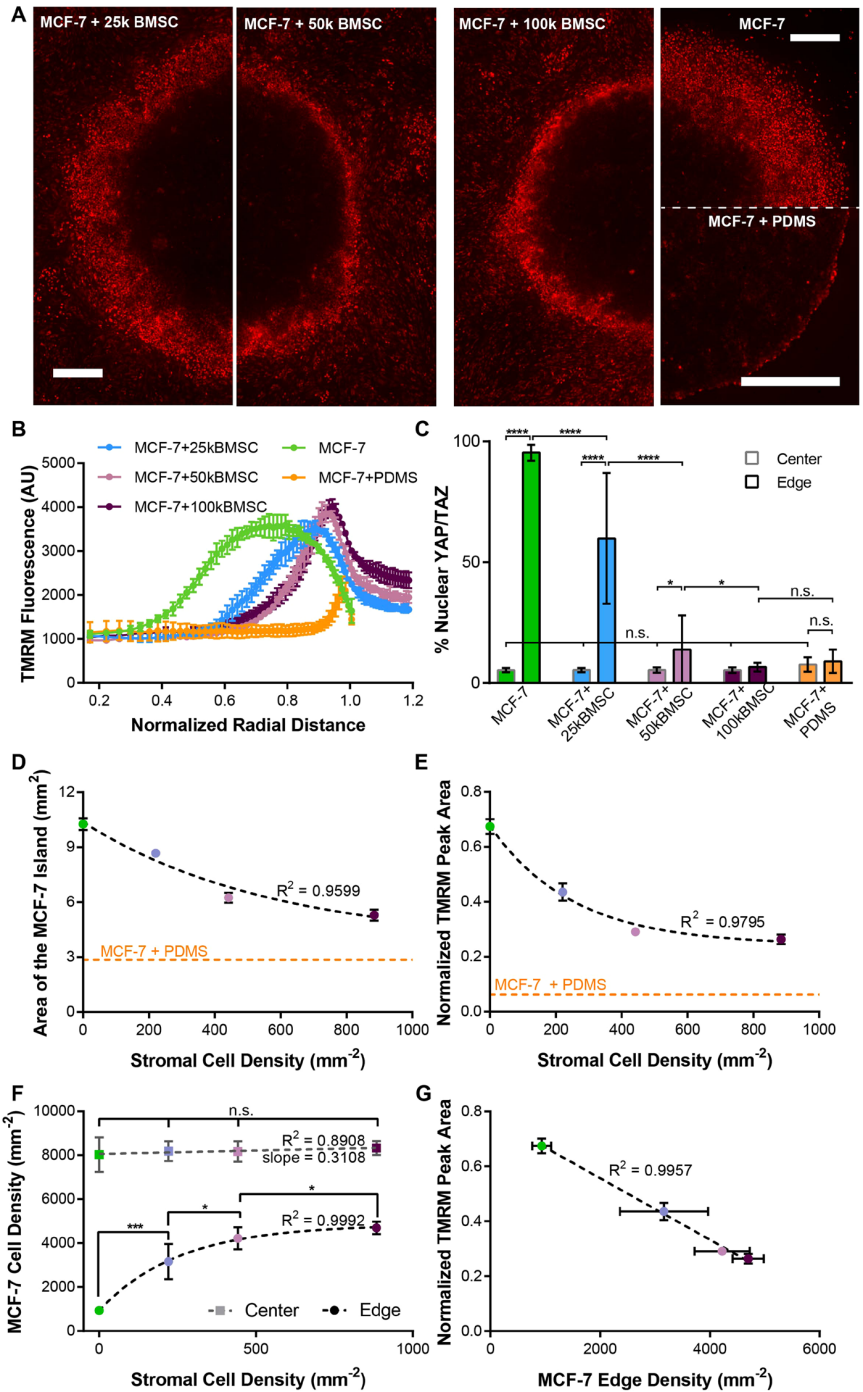
Regulation of cancer cell ΔΨ_m_ by stromal density. (**A**) TMRM fluorescence in a µTSA with BMSC seeding densities varying from 25,000, 50,000, to 100,000 cells per micropattern. Monocultures without or with PDMS constraint were used as controls. Scale bars: 500 μm; (**B**) Normalized radial distribution of TMRM fluorescence in micropatterns; (**C**) Percentage of YAP/TAZ nuclear localization in cancer cells at the edge and center of the micropatterns (n.s.: not significant, *p < 0.05, ****p < 0.0001 by ordinary one-way ANOVA); (**D**) Areas of cancer islands as a function of the initial stromal density on Day 4. Non-liner regression: single-exponential decay; (**E**) TMRM peak area at the interface normalized to total cancer area of the respective micropattern as a function of initial stromal density. Non-linear regression: single-exponential decay; (**F**) Cancer cell densities at the center (red curve) and edge (black curve) of micropatterns. (*p < 0.05, ***p < 0.001, by Kruskal-Wallis test); (**G**) Normalized TMRM peak area as a linear function of cancer cell density at the interface. Linear regression R^2^ = 0.9957. Representative dataset shown from N = 3 independent experiments for MCF-7, MCF-7 + 50k BMSC, and MCF-7 + PDMS micro-patterns, N = 1 for MCF-7 + 25k BMSC and MCF-7 + 100k BMSC micropatterns.

We noticed that the width of the cancer cell region with high ΔΨ_m_ at the tumor-stromal interface of the µTSA co-culture was variable within the same micropattern, as illustrated in Fig. 4B. A similar non-uniformity was also seen with nuclear translocation of YAP/TAZ in these co-cultures, as evidenced by the large standard deviation in Fig. 4C. As YAP/TAZ is involved in contact-mediated growth regulation, we hypothesized that these variations may be due to differences in local cancer cell densities as a result of differential stromal constraints. To test this hypothesis, we investigated whether the ΔΨ_m_ profile, YAP/TAZ nuclear translocation, and interfacial cancer cell density could be altered by the initial seeding density of the stromal cells. MCF-7 cells were micropatterned with three stromal densities (221, 442 or 884 cells/mm^2^, corresponding to 25k, 50k, and 100k initial seeding numbers in Fig. 5A–C), and stained for ΔΨ_m_ with TMRM and YAP/TAZ by immunostaining on day 4. Consistent with our hypothesis, we found that the interfacial region of cancer cells with high ΔΨ_m_ was much wider in the micropatterns with a lower stromal density than in those with higher densities (Fig. 5A). As controls, the open-edge and PDMS-confined mono-cultures showed the widest and narrowest regions of cancer cells with high ΔΨ_m_, consistent with those in Fig. 4. To quantitatively compare the ΔΨ_m_ profiles, we analyzed the radial distribution of TMRM fluorescence in the micropatterns. In the open-edge micropatterns, the cancer cell region with high ΔΨ_m_ was wide, with ΔΨ_m_ peaking rapidly at a normalized radial distance as low as 0.67 (the edge is at r = 1). In the µTSA co-cultures, however, the width of the high ΔΨ_m_ region was narrower and the peak closer to the interface (r = 0.85, 0.91, 0.92 as stromal cell seeding density increased) (Fig. 5B). In the PDMS-confined micropatterns, ΔΨ_m_ was uniformly low throughout the tumor island, except at the very edge, where cells clump along the PDMS barrier, leading to a slight increase in observed TMRM fluorescence. Strikingly, YAP/TAZ nuclear localization followed a similar trend as the ΔΨ_m_. The increased stromal density led to lower nuclear YAP/TAZ in the interfacial cancer cells (Fig. 5C). Noticeably, under the initial stromal density of 50k per well (442 cells/mm^2^), interfacial cancer cells still had significantly higher nuclear YAP/TAZ than those in the center. However, such difference was abrogated with higher stromal seeding density. Cells at the center of all the micropatterns showed a uniformly low nuclear YAP/TAZ localization (Fig. 5C).

Next, we quantitatively analyzed the impact of stromal densities on cancer cell growth and size, and their relationship with the ΔΨ_m_ profile. While cancer cells are generally considered not to be subjected to growth arrest from contact inhibition^51^, in our µTSA model, the area of the tumor island with higher stromal density appeared smaller than those seeded with lower stromal density (Fig. 5A,D), suggesting growth retardation and/or size restriction due to the physical constraints imposed by stromal cells. To simplify the analysis, we initially modeled cell growth in the micropatterns by measuring the size of the tumor islands. Cell growth is typically described as an exponential model, $Y={Y}_{0}{e}^{kt}$, where ${Y}_{0}$ is the initial population size, *k* is the growth rate, and *t* is the growth time^52^. To incorporate the impact of stromal density, we modified the growth curve to:

1 $$Y={Y}_{0}{e}^{k(s)t}$$

where $k(s)$ becomes a function of stromal density *s*. Interestingly, using curve fitting, we found a simple relationship of exponential decay between *Y* and *s* (R^2^ = 0.9599) (Fig. 5D). With growth time *t* being a fixed value (Day 4), the result implies that $k(s)$ is a linear function of *s* with a negative slope:

2 $$k(s)=-{k}^{\text{'}}s$$

Here, $k\text{'}$ is a rate constant representing the growth restricting effect from the stromal confinement. Interestingly, we found that the area of cancer cells with high ΔΨ_m_ (normalized by the total area of the tumor island) also had a similar relationship with stromal density, with a high coefficient of determination (R^2^ = 0.9865) (Fig. 5E), suggesting a direct role of physical confinement on ΔΨ_m_ distribution. We further investigated whether such regulation is mediated through the cancer cell size at the tumor-stromal interface. We plotted cancer cell densities at the center and interface of the µTSA against the initial stromal seeding densities. Indeed, higher stromal seeding density correlated with higher cancer cell density (thus smaller cancer cell sizes) at the edges of the micropatterns (Fig. 5F). Lastly, a strong negative correlation existed between the density of cancer cells and the normalized area of cancer cells with high ΔΨ_m_ (Fig. 5G). These results suggest that stromal confinement controls the spatial distribution of ΔΨ_m_ in cancer cells by regulating their growth and size.

### Inhibiting the Rho-associated protein kinase and actin polymerization leads to a loss of ΔΨ_m_ spatial distribution

Rho-associated protein kinase (ROCK) and the actin cytoskeleton are upstream of YAP/TAZ and are intricately involved in both contact-mediated Hippo-YAP signaling and contact-independent mechano-signaling through YAP/TAZ^49,53^. To investigate the involvement of ROCK and actin cytoskeleton in the spatial distribution of ΔΨ_m_, we used two chemical inhibitors, Y-27632 and Latrunculin A (LatA), both of which relax cellular actin tension and inhibit mechanotransduction^54,55^, and the open-edge monoculture (Fig. 4A, right), which eliminates the potential influence of stromal factors other than the physical confinement, while retaining the spatial distribution of ΔΨ_m_ (Fig. 4B, right). LatA directly inhibits actin polymerization, and Y-27632 decreases actin tension by phosphorylating myosin light chain and activating myosin II^56^. Both Y-27632 and LatA inhibited regular actin polymerization in MCF-7 cells at the center and edge of the micropatterns (Supplementary Fig. S1). At the center of the micropatterns, the MCF-7 cells originally had actin localization at the cell junctions, which became discontinuous upon treatment with Y-27632 and LatA. At the edge of the micropattern, those treated with Y27632 acquired thin lamellar extensions and showed a decrease in actin stress fibers, while those treated with LatA demonstrated an increased abundance of punctate actin clusters (Supplementary Fig. S1), as reported elsewhere^50,57^.

**Figure 6:**
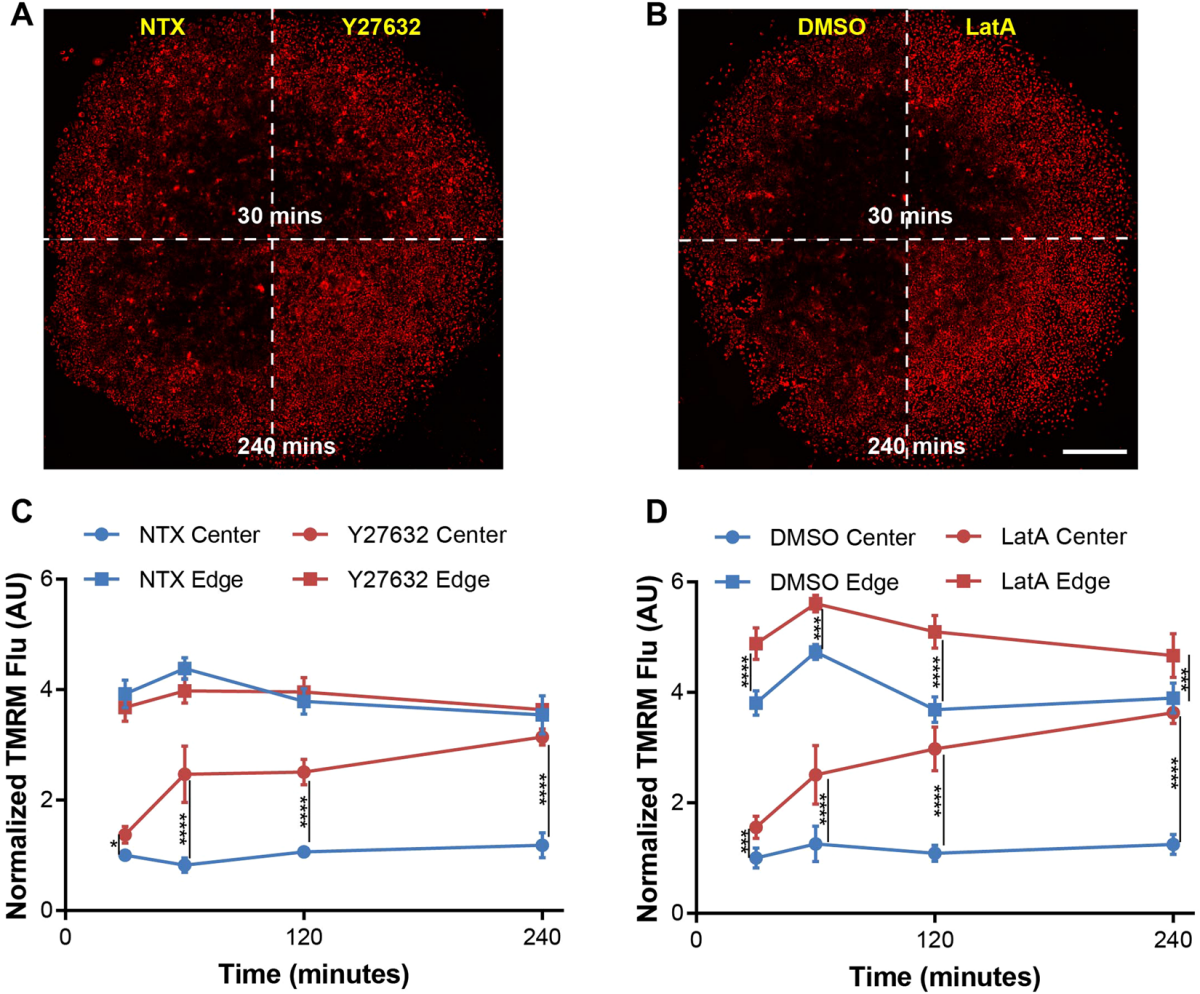
Loss of ΔΨ_m_ heterogeneity by inhibition of mechanotransduction. (**A**,**B**) TMRM fluorescence in monoculture µTSA on day 4 treated with inhibitors of mechanotransduction: Y-27632 (50 μM, ROCK inhibitor) and Latrunculin A (LatA, 0.5 μM, actin polymerization inhibitor) from 30 to 240 minutes. Scale bar: 500 μm; (**C**,**D**) Changes of TMRM fluorescence in cancer cells at the center and edge as a function of treatment time (Representative dataset from N = 3 independent experiments; *p < 0.05, ***p < 0.001, ****p < 0.0001, by ordinary one-way ANOVA).

As shown in Fig. 6A,B, both Y-27632 and LatA increased the ΔΨ_m_ (TMRM) in the cancer cells at the center of the micropatterns over a 4-hour period. The increase started as early as 30 min after treatment, rose rapidly between 30 min and 1 hour, and became slower from 1 to 4 hours (Fig. 6C,D). Notably, the two drugs had distinct effects on cancer cells. Y-27632 increased the ΔΨ_m_ only at the center of the micropatterns (Fig. 6C), while LatA treatment significantly enhanced the ΔΨ_m_ both at the edge and center of the micropatterns at all the time points (Fig. 6D). These results demonstrate a dependence of ΔΨ_m_ regulation on actin cytoskeleton^58^ and Rho-ROCK signaling^59^, and on cell location within the micropatterns.

### ΔΨ_m_ level correlates with metastatic potential *in vivo*

**Figure 7:**
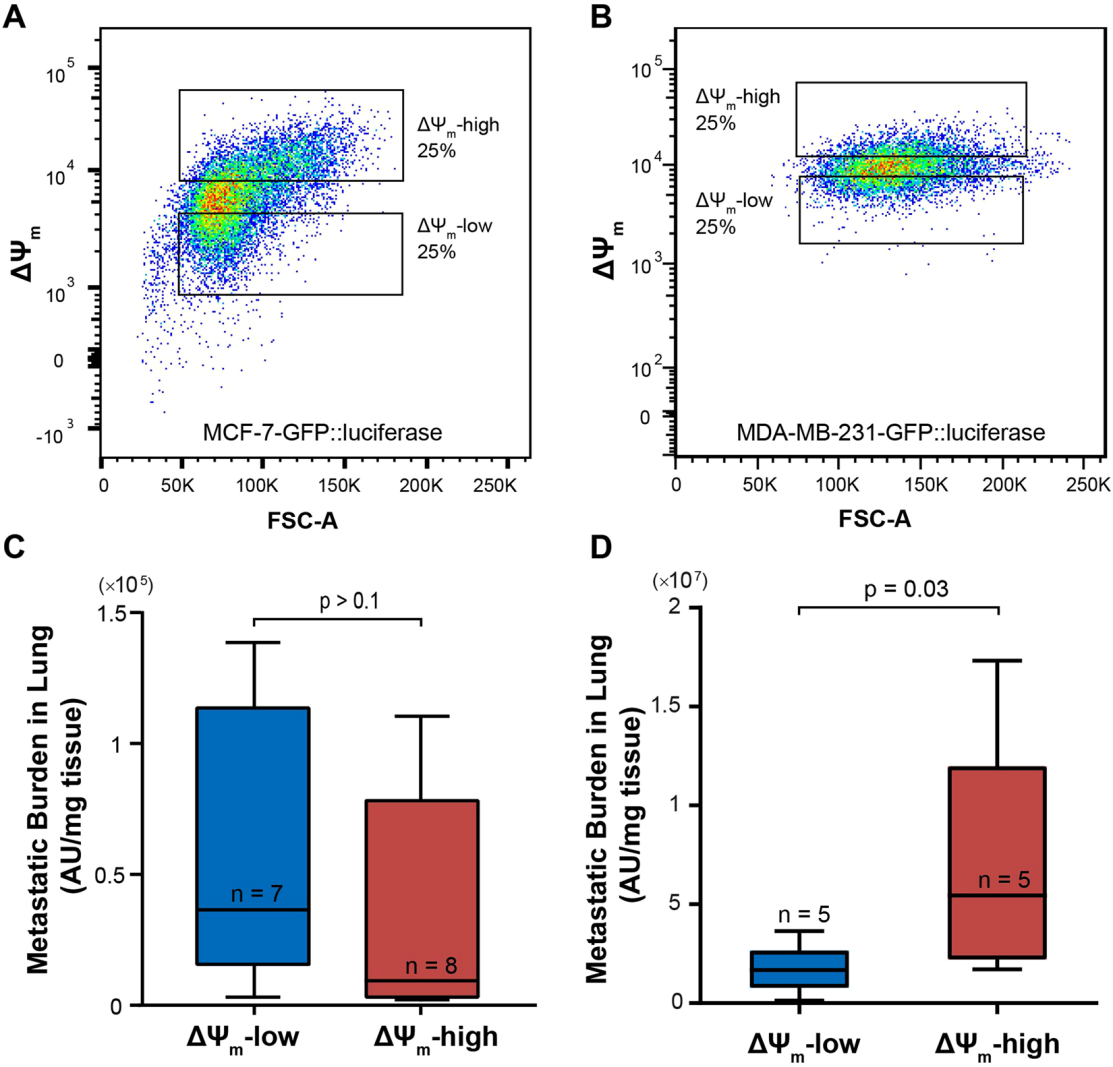
Correlation of ΔΨ_m_ with metastatic potential in vivo. (**A**) MCF-7 cells and (**B**) MDA-MB-231 cells, both transduced with GFP/luciferase, were sorted into a ΔΨ_m_-high and -low subpopulations for tail-vein injection into NSG mice; Quantification of the metastatic burden in the lungs of mice injected with (**C**) MCF-7 cells at week 5 and (**D**) MDA-MB-231 cells at week 4, via *ex vivo* quantification of luciferase activity in tissue lysate (p-values: Mann-Whitney test).

We next sought to investigate the *in vivo* significance of the ΔΨ_m_ heterogeneity. RNA-seq data suggested that cancer cells at the tumor-stromal interface have higher ΔΨ_m_ and elevated expression of a gene set related to metastasis (Fig. 1C). MCF-7 cells are known to be weakly metastatic^60,61^. Therefore, we used both MCF-7 and MDA-MB-231 cells, the latter of which is a metastatic breast cancer cell line, to determine if any correlation exists between ΔΨ_m_ and metastatic potential *in vivo*. MCF-7 and MDA-MB-231 cells were sorted into subpopulations with high and low-ΔΨ_m_ (top and bottom 25%, Fig. 7A,B), and then separately inoculated through tail vein injections into immune-deficient NSG (NOD.Cg-*Prkdc*^*scid*^ *Il2rg*^*tm1Wjl*^*/SzJ*) mice (the mice for MCF-7 cells were pre-implanted with estrogen pellets; see Methods). Both MCF-7 and MDA-MB-231 cells had been previously transduced with a GFP/firefly luciferase-reporter construct for downstream *ex vivo* measurement^62,63^. Metastases were allowed to develop for four (for MDA-MB-231) or five (for MCF-7) weeks post injection. At the end, mice were sacrificed, their lungs were snap-frozen, crushed, and lysed, and the luciferase signal per tissue weight was measured to assess metastatic burden^17^. In mice injected with MCF-7 cells, the luciferase signal from the lung tissues was low compared to those with MDA-MB-231 cells (Fig. 7C,D), and there was no significant difference between the lung metastatic burden between mice injected with ΔΨ_m_-high and those with ΔΨ_m_-low MCF-7 cells (Fig. 7C). Strikingly, while MDA-MB-231 cells are known to be highly aggressive, and had a tighter distribution of ΔΨ_m_ than MCF-7 cells (Fig. 7B), mice injected with the ΔΨ_m_-high MDA-MB-231 cells had a significantly higher metastatic burden than those injected with ΔΨ_m_-low cells (p = 0.03, Mann-Whitney test, Fig. 7D).

## Discussion

TME-mediated metabolic reprogramming plays an important role in sustaining tumor growth and progression^8,21,64–70^. In this study, by using a micropatterned co-culture model mimicking the morphological features of early breast tumors^17,18^, we show that mitochondrial and metabolic phenotypes can be spatially regulated by surrounding stromal cells under the architectural context of tumor-stromal interactions. Specifically, mitochondrial mass, ΔΨ_m_ and optical redox ratio (ORR) are enhanced near the tumor-stromal interface as compared to the center of the tumor nest. Importantly, ΔΨ_m_, a mitochondrial feature previously linked to cancer invasiveness^10^, is directly controlled by the physical confinement imposed by the surrounding stromal cells, which correlates with YAP/TAZ nuclear localization. We further demonstrated that the spatial regulation of ΔΨ_m_ by physical confinement is dependent on ROCK signaling and actin polymerization, and that high ΔΨ_m_ correlates with increased metastatic potential *in vivo*. To the best of our knowledge, such relationships have not been previously reported in cancer cells. Our results suggest that cancer cells can perceive physical cues from stromal cells, which may potentially regulate their metastatic behavior. As this study was done with breast cancer cells, more studies are needed to further examine the signaling pathways underlying the physical regulation of mitochondrial heterogeneity, and determine the applicability of these findings to other cancer and stromal cell types. Notably, some other physical cues within the TME, such as oxygen gradients and ECM stiffness, are also tightly associated with the tumor architecture^71,72^ in early cancer progression, and have been shown to alter cancer cell metabolism^73–75^. Additional studies are envisioned to elucidate the contribution of these components to the heterogeneous mitochondrial phenotypes.

In the current study, we uncovered that physical confinement from stromal cells controls the local density/size of the cancer cells and their ΔΨ_m_ level (Fig. 5). Interestingly, it has been reported that mitochondrial content/mass scales linearly with cell size in HeLa cells^76^, human umbilical vein endothelial cells (HUVECs)^77^, and budding yeast^78^. More recently, Miettinen and Bjorklund showed that ΔΨ_m_ and OXPHOS follow a non-linear, bell-shaped relationship with cell size, and those with an ‘optimal’ cell size at the highest ΔΨ_m_ seem to have decreased apoptosis and enhanced cellular proliferation^77^. Our observed co-upregulation of mitochondrial mass, ΔΨ_m_ and OXPHOS in the µTSA thus support the cell size theory and suggest a survival/proliferative advantage of the interfacial cancer cells in the µTSA. More intriguingly, our study further revealed an adaptive response of cancer cell size and ΔΨ_m_ to the physical confinement by stromal cells in TME. As stromal density increases, the density of cancer cells at the interface becomes higher; however, it plateaus at a density that is still much lower than that at the center of the micropattern (Fig. 5F). Nevertheless, while the width of the interfacial cancer cell region with higher ΔΨ_m_ becomes narrower with higher stromal density, the peak fluorescence intensity of the ΔΨ_m_ staining remains similar across all the patterns (except the PDMS confined culture). These results suggest that the cancer cells near the tumor-stromal interface can accommodate and adjust their sizes to retain an ‘optimal’ mitochondrial state/functionality, which is absent in the PDMS-confined culture.

In this study, we also observed preferential localization of YAP/TAZ in the cytoplasm or nuclei of cells at the center or interface of µTSA, respectively. This observation is in agreement with previous findings on YAP/TAZ regulation by cell density^53^. Importantly, at the population level, we further revealed a linear correlation between ΔΨ_m_ and YAP/TAZ nuclear translocation in the micropatterns (Fig. 4D). It is unclear, however, whether this represents a causal relationship. It has been shown that YAP overexpression in breast cancer cells leads to increased mitochondrial mass^79^. On the other hand, PLD6-mediated increase in mitochondrial fusion was shown to inhibit YAP/TAZ activity through AMPK-mediated YAP/TAZ phosphorylation in human mammary epithelial cells^80^. These studies and ours suggest a crosstalk between mitochondrial dynamics and YAP/TAZ activation. Additional studies are needed to further elucidate the signaling relationship between ΔΨ_m_ and YAP/TAZ.

It is noteworthy that nuclear YAP/TAZ have been found to act as relays of mechanical signals resulting from cell density^53^, ECM stiffness^49^, and intracellular cytoskeletal tension^56^. Recent studies have shown mitochondrial functions to be actin-dependent. Mitochondrial fusion and fission rely on dynamic actin polymerization^29^. ΔΨ_m_ cannot be maintained in mouse embryonic fibroblasts when β-actin is knocked out^81^. On the other hand, Furukawa *et al*. have recently shown that the cell density dependence of YAP/TAZ localization is mediated through intracellular actin tension^56^. Cellular actin tension was reduced in the micropatterns using LatA and Y-27632. The distinct changes in cell morphology and actin localization upon treatment with LatA and Y-27632 were consistent with previously reported results^50,57^. On the other hand, LatA and Y-27632 treatments did not lead to differences in nuclear YAP/TAZ, even after increasing treatment time to 12 hours (Supplementary Fig. S2). The lack of response of YAP/TAZ localization to both drugs is consistent with a previous report that the dependence of YAP/TAZ localization on F-actin integrity occurs only in the absence of cell-cell contacts, but not with confluent epithelial cells^50^. Both these drugs led to an increase in cellular ΔΨ_m_ in cancer cells at the center of the micropatterns (Fig. 6), confirming actin stress polymerization is upstream of ΔΨ_m_ regulation. Interestingly, at the edge of micropatterns, LatA treatment increased ΔΨ_m_ while Y-27632 has no effect, indicating a difference in the ΔΨ_m_ regulation in cancer cells between the edge and center of the micropattern. A possible explanation is the difference in the activation of YAP signaling in these two regions. Activated nuclear YAP has been shown to negatively regulate Rho^82,83^, and Rho activity is required to activate ROCK through its Rho-binding domain^84^. The difference in YAP/TAZ nuclear translocation between the two spatial regions could thus explain the difference in sensitivity to ROCK inhibition. The differential outcome with inhibition of cytoskeletal tension also suggests that cancer cells may experience different mechanical stresses (tensile vs. compressive) at the interface and center of the µTSA, which have been shown to associate with tumor architecture in a growing tumor mass^23^. Future studies aiming at measuring the distribution of stresses in the µTSA are envisioned.

Importantly, while our study points to the physical confinement imposed by the stromal cells as the main source of spatial ΔΨ_m_ distribution, it does not rule out other mechanisms that may also regulate mitochondrial functions, such as biochemical signaling through cell-cell adhesion and soluble factors. One such indication comes from the fact that the interfacial cancer cells under high stromal density do not behave as the PDMS-confined cancer cells (Fig. 5A,B). Interestingly, at the highest stromal seeding density (100k), YAP/TAZ was located in the cytoplasm both at the tumor-stromal interface and at the center (Fig. 5C); however, under the same conditions, the tumor-stromal interface still retains a thin layer of cancer cells with high ΔΨ_m_. One potential mechanism of this interface-bulk difference is the metabolic interactions between tumor and stromal cells, as well as between tumor cells from different micropattern regions. For instance, both cancer-associated fibroblasts (CAFs)^85^ and hypoxic tumor cells^86^ have been shown to undergo glycolysis and to supply lactate to fuel cancer cells that engage in mitochondrial OXPHOS. Notably, while there is no apparent hypoxia in the µTSA, cancer cells in the center have upregulated glycolytic gene expression compared to the interfacial cells (Fig. 1C). Another potential mechanism is through heterotypic tumor-stromal adhesion or cancer-ECM interactions at the epithelial-stromal interface. For example, breast cancer cells stimulate CCL5 production from BMSCs upon tumor-stromal contact^30^, which increases glucose uptake and ATP production in breast cancer cells^87^. Breast cancer cells can also secrete growth factors to activate nearby stromal cells^88^, which remodel the physical and biochemical properties of the surrounding ECM through matrix metalloproteinases^89^ and production of collagen^90,91^. Future studies are needed to determine the exact contributions of these biochemical factors to the heterogeneous mitochondrial phenotypes in µTSA.

While previous studies have reported association of high ΔΨ_m_ in cancer cells with increased secretion of vascular endothelial growth factor (VEGF) and matrix metalloproteinase-7 (MMP-7) as well as enhanced invasiveness *in vitro*^10^, the *in vivo* significance of ΔΨ_m_ in cancer metastasis remained unclear. Our RNA-seq data suggested increased expression of metastasis related genes in interfacial cancer cells (Fig. 1C), leading us to hypothesize that the ΔΨ_m_ of breast cancer cells is associated with their *in vivo* metastatic potential. One of the challenges of testing this hypothesis is that the MCF-7 breast cancer cells are only weakly metastatic *in vivo*^60,61^ and MCF-7 mouse models are generally considered to model early-stage breast cancer^92^. We inoculated mice with MCF-7 cells sorted based on their ΔΨ_m_ and assayed the lungs for metastatic burden five weeks post injections. Not surprisingly, the lung metastatic burden with MCF-7 cells was low despite of the higher inoculation doses than the MDA-MB-231 injection group. This is in accordance with a previous report which showed that MCF-7 cells injected through tail vein injection only resulted in scattered cancer cell presence in the lungs even after nine weeks, as opposed to large multicellular lung metastases in those injected with MDA-MB-231 cells in just two weeks post injection^61^. Further, we observed no difference in the lung metastatic burden between the groups injected with ΔΨ_m_ -high vs. ΔΨ_m_ -low MCF-7 cells (Fig. 7C), which could have resulted from the overall low metastasis forming ability of the MCF-7 cells. In stark contrast, we found that high ΔΨ_m_ MDA-MB-231 cells, when injected into mice, formed significantly more lung metastases than those injected with low ΔΨ_m_ cells. Together, these results suggest that while the ΔΨ_m_ may not be sufficient by itself to promote metastasis, it contributes to the metastasis formation in later stage cancer progression (as supported by the MDA-MB-231 metastasis model).

Some mitochondrial activities have been previously shown to be involved in the process of breast cancer metastasis *in vivo*. PGC1-α, a master regulator of mitochondrial biogenesis, was found to be essential for lung metastasis formation in mice^13^. We demonstrated, for the first time, that breast cancer cells (MDA-MB-231) with higher ΔΨ_m_ have greater metastatic potential *in vivo* (Fig. 7), which underscores the importance of investigating TME-mediated mechanisms governing mitochondrial heterogeneity. Further studies are also necessary to elucidate the relationship between mitochondrial biogenesis and ΔΨ_m_, and the regulatory role of ΔΨ_m_ in metastatic cascades, such as persistence in circulation, extravasation, and survival at the metastatic sites.

In summary, we have shown the impact of tumor-stromal interactions in inducing spatial heterogeneity of mitochondrial activities in the TME, with implications on the metastatic potential of cancer cells *in vivo*. We demonstrated that the differential ΔΨ_m_ of cancer cells is mainly controlled by physical confinement from stromal cells, correlates with YAP/TAZ nuclear translocation, and is mediated through the actin cytoskeleton. Our study provides new insights into the role of TME in the regulation of mitochondrial heterogeneity and cancer metastasis.

## Materials and Methods

### Cells

MCF-7 and MDA-MB-231 cells (ATCC) were cultured in Dulbecco’s Modified Eagle Medium (DMEM; Life Technologies) supplemented with 10% Fetal Bovine Serum (FBS; EMD Millipore), and 100 U/ml penicillin and 100 µg/ml streptomycin (Life Technologies). Primary human bone marrow stromal cells (BMSCs) derived from whole human bone marrow aspirates (Lonza)^93^ were expanded using the MesenCult Proliferation Kit (Stem Cell Technologies). µTSA co-cultures were carried out in the FBS and penicillin/streptomycin supplemented DMEM.

### Antibodies

The following primary antibodies were used in this study: TOM20 (Santa Cruz Biotechnology, sc-17764, 1:50), YAP/TAZ (Cell Signaling, rabbit mAb 8418, 1:200), pan-keratin (Cell Signaling, C11 mouse mAb 4545, 1:500), and vimentin (Cell Signaling, rabbit mAb 5741, 1:200). Alexa Fluor-conjugated secondary antibodies (Life Technologies) were used at a dilution of 1:500.

### Micropatterned Tumor-Stromal Assay (μTSA)

Collagen-I was extracted from rat-tails (acetic acid extraction)^94^ and quantified using the modified Lowry method^95^. Glass coverslips (12 mm) were cleaned with 7X cleaning solution (MP Biomedicals), treated with plasma, silanized with 3-aminopropyltriethoxysilane (APTES), and coated with 0.1 mg/ml extracted collagen. A laser engraver (Epilog) was used to print stencils on 250 µm thick silicone sheets (PDMS), which were then successively cleaned in 70% isopropyl alcohol and Milli-Q water, and air-dried. Cleaned stencils were then aligned on top of collagen-coated coverslips, and this assembly was treated with 0.2% pluronic F-127 (Sigma) for 15 minutes, followed by PBS and DMEM rinses. The stencil-overlaid coverslips were placed in 24-well plates. MCF-7 cells were seeded at a density of 300,000 cells per micropattern in the 24-well plates and allowed to adhere onto the circular islands within the micropatterns for 5 hours. Next, the micropatterns were rinsed in DMEM to remove excessive cancer cells. After overnight incubation, stencils were removed from the coverslips and micropatterns were rinsed thoroughly in DMEM. BMSCs were seeded (at densities ranging from 25,000 to 100,000 cells per micropattern) and rinsed off from the cancer island by thorough DMEM rinsing within 30 minutes of seeding to allow for adhesion in the surrounding areas without contaminating the cancer cell region (day 0). The micropatterned cells were cultured for 4 days before the subsequent analysis.

### Imaging of the mitochondrial membrane potential (ΔΨ_m_) in the μTSA

Day 4 micropatterns were stained at 37 °C for 30 minutes with the mitochondrial membrane dye, tetramethylrhodamine methyl ester, (30 nM in complete medium; TMRM, Life Technologies). Following incubation, micropatterns were rinsed and immediately imaged in PBS using a Nikon Eclipse Ti inverted fluorescence microscope.

### Immunofluorescence

Day 4 micropatterns were fixed with 4% paraformaldehyde (Electron Microscopy Sciences) at room temperature for 15 minutes, rinsed with PBS, and permeabilized with 0.1% Triton X-100 (Fisher Scientific) for 10 minutes. Micropatterns were then blocked with 4% bovine serum albumin (GE Healthcare), either for one hour at room temperature or overnight at 4 °C, followed by incubation with the primary antibody for 2 hours at room temperature, three PBS rinses and incubation with the respective secondary antibodies at room temperature for 1 hour. Micropatterns were then rinsed three times with PBS and once with milli-Q water, mounted onto glass slides using FluoroGel II (Electron Microscopy Sciences), and imaged with the Nikon Eclipse Ti inverted microscope and/or the Nikon confocal microscope.

### Metabolic imaging (NAD(P)H and FAD fluorescence)

A Zeiss LSM-780 inverted confocal microscope coupled to a Ti-Sapphire laser system and an A320 FastFLIM FLIMbox^96^ was used to determine the fluorescence intensities of NADH and FAD in day 4 MCF-7/BMSC μTSA. Image processing and quantification were performed using a customized code in Python2.7.

### Laser capture microdissection (LCM) and RNA sequencing

Day 4 micropatterns were fixed in 100% ethanol, serially rehydrated in 75% ethanol and water, and stained with the Histogene Staining Solution (Applied Biosystems). The samples were then serially rinsed and dehydrated in water, 75%, 95%, and 100% ethanol, submerged in xylene, and air-dried immediately before being mounted onto glass slides with cell side up for LCM. The ArcturusXT Laser Capture Microdissection System was used to extract MCF-7 cells at the centers and edges of the MCF-7-BMSC µTSA. RNA from the micro-dissected cells was extracted using the PicoPure RNA Isolation Kit (Arcturus) following the manufacturer’s instructions. RNA concentrations were determined by absorbance on a NanoDrop One spectrophotometer and Qubit (ThermoFisher). RNA integrity was determined using the Agilent 2100 Bioanalyzer and RNA 6000 Nano Chip kit. Only samples with an RNA Integrity Number (RIN) score greater than 7 were used. Following mRNA purification from total RNA using poly-A selection, a cDNA library was prepared using Illumina TruSeq Stranded mRNA Library Prep kit. The cDNA library quality was checked using the Agilent 2100 Bioanalyzer and D1000 DNA Chip kit. Samples were run on the Illumina NextSeq500 platform using the High Output Sequencing Kit v2 (150 cycles, 2 × 75 bp read length, 20 million reads per sample). Library preparation and sequencing were performed by the Single Cell, Sequencing, and CyTOF (SC^2^) Core (Children’s Hospital of Los Angeles, Los Angeles, CA).

### Gene set enrichment analysis (GSEA)

Before analysis, raw FASTQ files from RNA-sequencing were checked for read quality using FASTQC. Next, reads were mapped to the most recent *Homo sapiens* reference genome (GRCh38) using the HISAT2 splice aligner^97^. After alignment, mapped reads were counted with R using the Rsubread Bioconductor package^98^. Read counts were arranged into a count matrix and differentially expressed gene (DEG) analysis was performed using DESeq. 2 in R^99^. A pairwise comparison using the Wald Chi-Squared test was employed to contrast gene expression within our two groups (μTSA interface versus center) and genes were subsequently ranked based on comparison significance (−log_10_(nominal p-value)*LFC direction; where LFC is the log fold change). GSEA was then conducted under default settings to identify the coordinated enrichment of functionally linked genes^100,101^. Briefly, the GSEA algorithm walks through the pre-ranked dataset and calculates a running enrichment score (ES) by increasing the ES when a dataset gene is found to be a member of the gene set of interest and decreasing the ES if it is not. A gene set is identified as enriched when the final ES is high and weighted towards a single phenotype. Statistical significance is determined by comparing the ES to the ES_null_ obtained by permutating the ranked dataset 1000 times and recalculating the ES. Nominal p-values are then adjusted for multiple testing correction through the false discovery rate (FDR) Benjamini-Hochberg method^102^. Gene sets with an FDR < 0.25 were considered as significantly enriched. Functionally linked gene sets were obtained from the Hallmark^103^, KEGG, and Gene Ontology^104^ collections compiled in Molecular Signatures Database (MSigDB)^100^.

### Mechanical constraints

A microcontact printing approach was used to create a PDMS barrier for the complete mechanical constraint in µTSA. To create a mold for the stamps, circular outlines (2 mm in diameter) were cut on a 140 µm thick protective film tape (Patco) with a desktop craft cutter (Silhouette Cameo). The circular tape cutouts were then transferred onto the bottom of 150 mm diameter petri dishes, and covered with a 1 mm-thick layer of PDMS (curing agent:base = 1:10, Sylgard 184, Dow Corning), degassed, and cured at 65 °C. The cured PDMS layer was then cut with a biopsy punch (10 mm in diameter) to obtain PDMS stamps with the 2 mm concave circular region at their center replica-molded from the tape cutouts. To create a PDMS barrier, a thin layer of freshly mixed and degassed PDMS was spin-coated on a round 18 mm-diameter glass coverslip. A stamp was placed on top of the thin PDMS layer, inked with PDMS, and then transferred onto a collagen-coated coverslip. The stamp was then removed and discarded. The transferred PDMS layer was cured overnight at room temperature. Before use, the coverslips were incubated in 0.2% pluronic F-127 (Sigma) for 15 minutes followed by rinses with PBS and DMEM. MCF-7 cells were then seeded to create completely confined tumor micropattern.

### *In vivo* metastasis model

All animal studies were carried out in accordance with the recommendations in the Guide for the Care and Use of Laboratory Animals of the National Institutes of Health. The protocol was approved by the Institutional Animal Care and Use Committee of the University of Southern California. MCF-7 and MDA-MB-231 breast cancer cells (ATCC), stably transduced with a GFP/luciferase construct^62,63^, were stained with TMRM or DiIC1(5) (MitoProbe DiIC1(5) Assay Kit, Invitrogen) as a marker of mitochondrial membrane potential and sorted on the BD FACSAria I cell sorter (Flow Cytometry Core Facility, USC). Sorted cells were inoculated into NSG (NOD.Cg-*Prkdc*^*scid*^ *Il2rg*^*tm1Wjl*^*/SzJ*) mice through tail vein injections (300,000 cells per mouse for MCF-7 and 100,000 cells per mouse for MDA-MB-231). For the MCF-7 study, mice were implanted with 17β-estradiol 90-day release pellets (0.36 mg/pellet), using a sterile trochar 24 hours prior to the injections. After five weeks (for MCF-7 cells) or four weeks (for MDA-MB-231 cells), the animals were euthanized and their lungs were harvested, ground, and assayed for metastatic tumor content using a Luciferase Assay System (Promega)^17^.

### Statistical analysis

Other than the RNA sequencing data (see above), all data are presented as mean ± standard deviation (SD), as stated in the figure legends. Statistical significance was assessed using the Welch’s t-test (parametric) and Mann-Whitney (non-parametric) for pair-wise comparison, and ordinary 1-way ANOVA for comparison between multiple (≥3) conditions; p < 0.05 was considered as significant.

## Supplementary information

Supplementary Information

## Data Availability

The raw RNA sequencing data files generated during the present study have been deposited in the NCBI Sequence Read Archive (SRA Bioproject ID PRJNA523041).
