# Supplementary material for: Spatial Regulation of Mitochondrial Heterogeneity by Stromal Confinement in Micropatterned Tumor Models
Source: Sci Rep. 2019 Aug 1;9:11187. doi: 10.1038/s41598-019-47593-8 (PMC6671984; doi:10.1038/s41598-019-47593-8)
Supplement: Supplementary file 1 — Supplementary Information [file 41598_2019_47593_MOESM1_ESM.pdf]

Supplementary Information

Spatial Regulation of Mitochondrial Heterogeneity by Stromal Confinement in Micropatterned Tumor Models

Hydari Masuma Begum[1], Hoang P Ta[1], Hao Zhou[1], Yuta Ando[1], Diane Kang[2], Kristen Nemes[3], Chelsea Mariano[1], Jia Hao[1], Min Yu[2,4], Keyue Shen[1,4,5*]

[1]Department of Biomedical Engineering, Viterbi School of Engineering, University of Southern California, Los Angeles, CA 90089

[2]Department of Stem Cell Biology and Regenerative Medicine, Keck School of Medicine, University of Southern California, Los Angeles, CA 90033

[3]Mork Family Department of Chemical Engineering, Viterbi School of Engineering, University of Southern California, Los Angeles, CA 90089

[4]Norris Comprehensive Cancer Center, Keck School of Medicine, University of Southern California, Los Angeles, CA 90033

[5]USC Stem Cell, Keck School of Medicine, University of Southern California, Los Angeles, CA 90033

*Correspondence should be addressed to Keyue Shen (keyue.shen@usc.edu)

Supplementary Figures

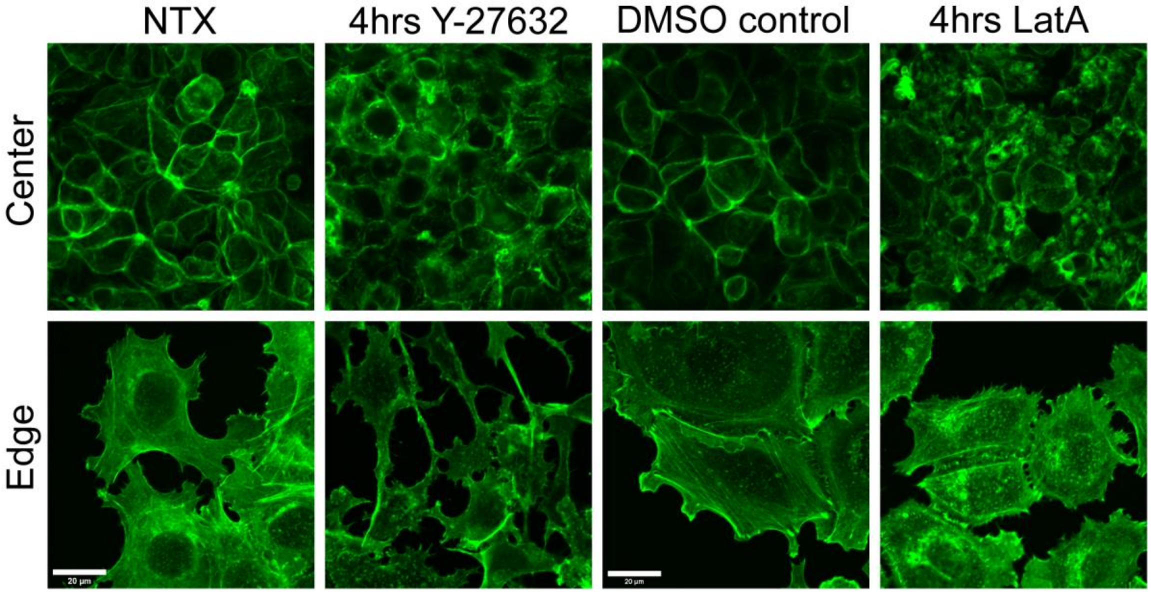

Figure S1. Fluorescence images showing F-actin staining in MCF-7 cells at the centers and edges of day 4 micro-patterns, after 4 hours of drug treatment with 50μM Y-27632 or 0.5μM Latrunculin A, along with their respective no treatment controls. Representative images shown from three images per condition, scale bar = 25μm.

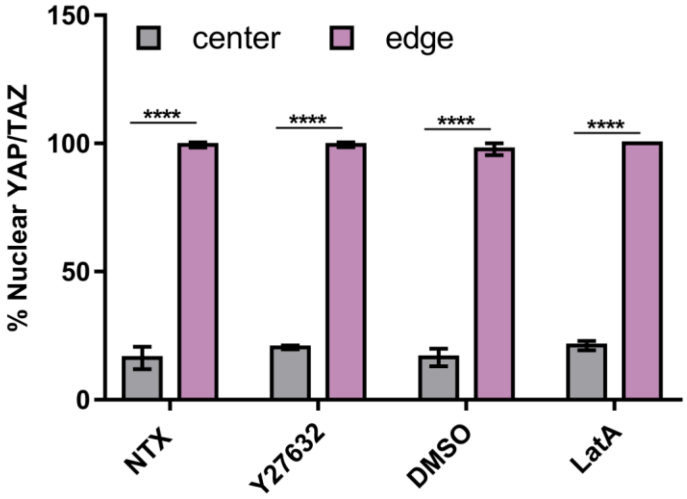

Figure S2. Quantification of nuclear YAP/TAZ in MCF-7 cells at the centers and edges of day 4 micropatterns, following 12 hours of drug treatment with 50μM Y-27632 or 0.5μM Latrunculin A, along with their respective no treatment controls. Three fluorescence images per region were quantified, $p < 0.0001$ in an ordinary One-Way ANOVA.
